# Alkylated epidermal creatine kinase as a biomarker for sulfur mustard exposure: comparison to adducts of albumin and DNA in an in vivo rat study

**DOI:** 10.1007/s00204-021-03005-3

**Published:** 2021-02-26

**Authors:** Dirk Steinritz, Robin Lüling, Markus Siegert, Julia Herbert, Harald Mückter, Christian D. Taeger, Thomas Gudermann, Alexander Dietrich, Horst Thiermann, Harald John

**Affiliations:** 1 Bundeswehr Institute of Pharmacology and Toxicology, Neuherbergstraße 11, 80937 Munich, Germany; 2 Walther-Straub-Institute of Pharmacology and Toxicology, Ludwig-Maximilians-Universität Munich (LMU), Goethestraße 33 / Nussbaumstraße 26, 80366 Munich, Germany; 3 Department of Plastic-, Hand- and Reconstructive Surgery, University Hospital Regensburg, Franz-Josef-Strauß-Allee 11, 93053 Regensburg, Germany; 4 Present Address: Bundeswehr Medical Service Academy, Ingolstädter Straße 240, 80939 Munich, Germany; 5 Present Address: Proteros Biostrucures GmbH, Bunsenstraße 7a, 82152 Planegg, Germany; 6 Present Address: Department of Pharmacology and Toxicology, Ernest Mario School of Pharmacy, Rutgers University, Piscataway, NJ USA

**Keywords:** Chemical warfare agents, Micro liquid chromatography, High-resolution tandem-mass spectrometry, Human skin

## Abstract

Sulfur mustard (SM) is a chemical warfare agent which use is banned under international law and that has been used recently in Northern Iraq and Syria by the so-called Islamic State. SM induces the alkylation of endogenous proteins like albumin and hemoglobin thus forming covalent adducts that are targeted by bioanalytical methods for the verification of systemic poisoning. We herein report a novel biomarker, namely creatine kinase (CK) B-type, suitable as a local biomarker for SM exposure on the skin. Human and rat skin were proven to contain CK B-type by Western blot analysis. Following exposure to SM ex vivo, the CK-adduct was extracted from homogenates by immunomagnetic separation and proteolyzed afterwards. The cysteine residue Cys^282^ was found to be alkylated by the SM-specific hydroxyethylthioethyl (HETE)-moiety detected as the biomarker tetrapeptide TC(-HETE)PS. A selective and sensitive micro liquid chromatography-electrospray ionization high-resolution tandem-mass spectrometry (µLC-ESI MS/HRMS) method was developed to monitor local CK-adducts in an in vivo study with rats percutaneously exposed to SM. CK-adduct formation was compared to already established DNA- and systemic albumin biomarkers. CK- and DNA-adducts were successfully detected in biopsies of exposed rat skin as well as albumin-adducts in plasma. Relative biomarker concentrations make the CK-adduct highly appropriate as a local dermal biomarker. In summary, CK or rather Cys^282^ in CK B-type was identified as a new, additional dermal target of local SM exposures. To our knowledge, it is also the first time that HETE-albumin adducts, and HETE-DNA adducts were monitored simultaneously in an in vivo animal study.

## Introduction

Sulfur mustard (SM, *bis*(2-chloroethyl) sulfide, CAS No. 505-60-2) is an internationally banned chemical warfare agent. The Chemical Weapons Convention, that is implemented by the Organisation for the Prohibition of Chemical Weapons (OPCW) (www.opcw.org/chemical-weapons-convention), prohibits its development, production, otherwise acquisition, stockpiling or retaining, transfer, and deployment. Nevertheless, SM has repeatedly been used in Northern Iraq and Syria by the so-called Islamic State (Quillen 2016) as reported by the OPCW in 2015 in Marea (Organisation for the Prohibition of Chemical Weapons 2015) or Kilic et al. who treated patients that were supposed to be exposed to SM in 2016 in Al-Bab (Kilic et al. 2018). Such alleged use of SM represents a serious violation of international law and requires definite evidence. For this purpose, bioanalytical methods are available to verify poisoning by the detection of unambiguous SM-induced covalent modifications of endogenous biomacromolecules, e.g. adducts of DNA and proteins (Gandor et al. 2015; John et al. 2016; Zubel et al. 2018, 2019; Noort et al. 2008). Such protein-adducts were shown to exhibit a half-life of several weeks thereby allowing sampling of plasma and serum even days or weeks after the event (Noort et al. 1996, 1999; Black et al. 1997; Xu et al. 2014; John et al. 2019; Steinritz et al. 2016). Human serum albumin (HSA), alkylated by SM at the only free cysteine residue (Cys^34^) by attaching a hydroxyethylthioethyl-moiety (HETE), is a frequently used protein-adduct to prove exposures (Noort et al. 1999; Gandor et al. 2015). In general, the use of HETE-HSA as biomarker is attractive because it can be assessed in human plasma which is easy to obtain. However, the uptake of SM into the circulation is a prerequisite for the formation of this adduct. Thus, the systemic biomarker generation depends on sufficient SM doses that must be absorbed by inhalation or by penetration of the skin. Especially when delivered as a liquid, the skin is the primary portal of entry for SM. The lipophilicity of SM combined with the affinity of the skin for lipophilic substances promote its uptake into the epidermis. However, only a small fraction of dermally applied SM seems to penetrate. In vitro experiments conducted by Riviere et al. revealed a resorption of only less than 5% (Riviere et al. 1995). This loss might be caused by e.g. evaporation processes on the warm skin, retention in lipophilic tissue and covalent binding to tissue components like proteins and DNA. Approximately 12% of a single SM dose reacts with components in the skin, most presumably within the epidermis (Renshaw 1946). Thus, skin exposures with small amounts of liquid SM may not result in systemic biomarker formation due to the limited dermal absorption. Hence, an exposure may not be detected when systemic biomarkers are used for verification in this scenario. This indicates the need for appropriate non-systemic dermal biomarkers.

Methods detecting SM-induced covalent modifications of DNA bases in the epidermis are available (Zubel et al. 2018, 2019). Especially mass spectrometry (MS)-based methods require tissue preparation, partial hydrolysis and isolation of the epidermal DNA. Epidermal skin is estimated to contain only 3 ng DNA per 1 µg tissue (Krämer et al. 1971). This low concentration either limits the chance for successful forensic analysis or requires punching of larger skin samples. Approaches addressing alternative, high abundant biomarkers may close this gap. As reported previously by our group, SM targets the reactive cysteine 282 residue (Cys^282^) in human creatine kinase (CK) adding the HETE-moiety (Lüling et al. 2020). Moreover, CK is present in the epidermis (Schlattner et al. 2002). Both aspects suggest the use of CK as a non-systemic, dermal biomarker to prove SM exposures. In the present study we investigated the potential of alkylated CK as a biomarker using the following approach: initially, the expression of CK in rat and human skin was shown by Western blot analysis. Next, rat and human skin samples were exposed *ex vivo* to SM followed by the extraction of CK applying immunomagnetic separation (IMS). Afterwards, micro liquid chromatography-electrospray ionization high-resolution tandem-mass spectrometry (µLC-ESI MS/HRMS) was used to identify the adduct formation at Cys^282^ of human and rat CK ex vivo. Finally, an *in vivo* animal study was conducted to evaluate the suitability of the HETE-CK biomarker and compare it to the already established HETE-albumin- and HETE-DNA-derived biomarkers.

## Materials and methods

### Chemicals

SM was provided by the German Ministry of Defense. Integrity and purity (at least 99%) were shown by in-house nuclear magnetic resonance (NMR) spectroscopy. Decontaminant (reactive skin decontamination lotion, RSDL) was also provided by the German Ministry of Defense. Alkaline phosphatase, benzonase, DNase, iodoacetamide (IAA), dimethyl pimelimidate (DMP), Tween20, phosphate-buffered saline (PBS), Tris base (TRIS), triethanolamine (TEA) buffer, ethanol (EtOH), benzonase, sodium acetate (NaOAc) and alkaline phosphatase (AP) were purchased by Sigma-Aldrich (Steinheim, Germany). Thiourea, urea, 3-[(3-cholamidopropyl)dimethylammonio]-1-propanesulfonate (CHAPS), protein inhibitor mix, nuclease mix, 2D-Quant Kit for protein quantification and dithiothreitol (DTT) were provided by GE Healthcare (Chicago, IL, USA). Phosphodiesterase (PDE), Dynabeads™ Protein G, 4–12% bis–tris gels, NuPAGE SDS MES running and transfer buffers, and polyvinylidene fluoride (PVDF) membranes were obtained from Thermo Fisher Scientific (Waltham, USA). Proteinase K (ProtK), water (LC–MS-grade), formic acid (FA, 98–100%) and MgCl_2_ were delivered by Merck (Darmstadt, Germany). Triple-deuterated atropine (d_3_-atropine) was purchased from CDN Isotopes (Pointe-Claire, Quebec, Canada) and NH_4_HCO_3_ (ultra-grade, ≥ 99.5%) from Fluka (Buchs, Switzerland). HCl, NaCl and NaN_3_ were obtained from Carl Roth (Karlsruhe, Germany). Rabbit monoclonal anti-CK B-type, mouse monoclonal anti-CK M-type and mouse monoclonal anti-CK MB-type antibodies were purchased from Abcam (Cambridge, UK). As secondary antibodies, IRDye® 800CW goat anti-rabbit IgG and IRDye® 800CW goat anti-mouse IgG were used (LI-COR Biosciences, Bad Homburg, Germany).

### Skin origin for ex vivo experiments

Ratskin flaps were derived from study-independent ex vivo or in vivo experiments (all approved by the animal welfare commission) and were kindly provided by the Bundeswehr Institute of Pharmacology and Toxicology (Munich, Germany). All human skin samples were taken from resectates that were obtained during operations and would otherwise have been discarded. This was approved by the ethics commission of the university hospital of Regensburg.

### Exposure of rat and human skin to SM ex vivo

Skin biopsies with a diameter of 8 mm (approximately 50 mm^2^) were taken from ex vivo rat or human skin using a circular blade biopsy tool (DocCheck Shop, Cologne, Germany). Specimens were transferred into separate glass vials, aligned upwards with the epidermis facing to the top and 10 µL of either neat SM (12.7 mg) or of different ethanolic SM solutions (corresponding to absolute amounts of 0.00127, 0.0127, 0.127, and 1.27 mg SM) were added. The respective SM solution was applied as a single drop precisely on top of the epidermis, without additional contact of any other parts of the specimen. To generate blank samples, 10 µL EtOH was used instead of SM. After 1 h incubation at room temperature (RT) under a fume hood, 100 µL RSDL was added to each vial to allow decontamination. After 15 min incubation, RSDL was discarded and samples were rinsed with deionized water. Each vial was gently agitated, and the supernatant was discarded. This procedure was repeated three-times before 500 µL deionized water was added prior to homogenization. Exposure experiments were carried out using three independent skin samples (*n* = 3) for each concentration.

### Homogenization of skin, lysis, and determination of protein concentration

The water covering the skin biopsies was removed and 1 mL lysis buffer (7 M urea, 2 M thiourea, 4% w/v CHAPS, 40 mM DTT, 10 µL proteinase-inhibitor mix, pH 8.5) was added to each sample. Skin samples were homogenized using an T25 digital ultra-turrax system equipped with an 8 mm diameter dispersion tool (IKA®-Werke, Staufen, Germany). The glass vials containing the biopsies were placed on ice and subjected to 8 cycles with 10 s homogenization time at a maximum rotation speed. The tissue debris was removed by centrifugation for 20 min at 4 °C and 2,500 RCF. Supernatants were transferred into a reaction vial and 5 µL nuclease mix was added. Samples were then incubated for 60 min at RT. Still undissolved particles were discarded after centrifugation (30 min, 4 °C, 21,130 RCF) and subsequent transfer of the supernatant into another reaction vial. Protein concentration was determined using the 2D-Quant Kit according to the manufacturer's instructions. Samples were frozen at − 20 °C afterwards prior to further analysis.

### Western blot analysis

A portion of the final protein solution (Sect. Homogenization of skin, lysis, and determination of protein concentration) containing 10 µg protein was taken from each sample. After adding 2 µL sample loading buffer (LI-COR Biosciences) and 6 µL aqueous DTT solution (80 mg/mL), water was added to obtain a final volume of 25 µL. Samples were boiled at 95 °C for 5 min to reduce and denature the proteins. Afterwards, 10 µg protein was loaded per lane on precast polyacrylamide gel electrophoresis (PAGE) gradient gels (4–12% *bis*–tris gel). A molecular weight marker (Chameleon Duo pre-stained protein ladder 8–260 kDa, LI-COR Biosciences) was loaded to one lane per gel. SDS-PAGE in NuPAGE SDS MES running buffer was performed for 45 min at 180 V. Afterwards, proteins were blotted onto a PVDF membrane that was activated with methanol for 1 min before. The wet transfer was performed in a mini blot module (ThermoFisher Scientific) for 1 h at 20 V using NuPAGE transfer buffer. The PVDF membrane was blocked with PBS blocking buffer (LI-COR Biosciences) for 1 h at RT. Membranes were incubated overnight at 4 °C with either anti-CK B-, anti-CK M- or anti-CK MB-type antibodies diluted 1:1,000 in PBST (0.2% v/v Tween20 in PBS). Afterwards, membranes were washed three times for 10 min each with PBST, and then appropriately incubated with either goat-anti-rabbit 800CW or goat-anti mouse 800CW secondary antibody (1:15,000 dilution of 1 mg/mL in PBS blocking buffer with 0.2% v/v Tween20) for 1 h at RT. After three times washing with PBST, blots were scanned on a LI-COR odyssey scanner (LI-COR) in automatic mode.

### Purification of CK from skin lysates by IMS and proteolysis

A portion of 500 µL of the commercially available magnetic Dynabead slurry was transferred into a reaction vial to remove the liquid layer on a magnet stand. Beads were washed with 1 mL PBST for three times. Afterwards, beads were incubated with 140.8 µL anti-CK B-type antibody solution in PBST (0.71 mg/mL) and 2 mL PBST for 15 min at RT on a nutating mixer. After removal of the supernatant, beads were washed twice with 1 mL TEA buffer (200 mM TEA and 0.025% v/v NaN_3_ dissolved in water). Finally, 1 mL DMP-solution (5.4 mg/mL in TEA buffer) was added. The mixture was incubated for 30 min at RT on a nutating mixer. The supernatant was removed, and 1 mL TBS (200 mM Tris–HCl, 9% w/v NaCl) was added for a 15 min incubation. After two washing steps with 500 µL PBST, beads were suspended in 475 µL PBS and stored at 4 °C.

For each IMS step, 50 µL of the labeled bead suspension in PBS was incubated with 2 mg protein sample for 24 h at 4 °C on nutating mixer. Afterwards, the supernatant was removed, and beads were washed twice with 500 µL PBST. Beads were suspended in 100 µL IAA-solution (40 mM IAA in HPLC-water) for 30 min and washed twice with 500 µL PBST. After removing the washing solution, 10 µL ProtK solution (15 mg/mL in 50 mM NH_4_HCO_3_) and 75 µL water were added to incubate for 2 h at 50 °C in a thermal mixer. The liquid phase was transferred into an ultrafiltration (UF)-device with a molecular weight cut-off (MWCO) of 10 kDa (Vivaspin 500 centrifugal concentrator, Sartorius Stedim, Göttingen, Germany) and centrifuged for 10 min at 10,000 RCF. A portion of 100 µL d_3_-atropine solution (3 ng/mL in 0.5% v/v FA) was added as internal standard and samples were ultrafiltrated for 10 min at 10,000 RCF. The filtrate was transferred into a glass vial and stored at 4 °C for MS analysis.

### In vivo animal study

Male Wistar rats (9–10 weeks old, weighing 275–300 g) were obtained from Charles River Germany (Sulzfeld, Germany). Animals were kept under standard housing conditions, with food and water ad libitum, controlled temperature/humidity and a 12/12 h light/dark cycle. All experimental procedures were approved by the commission for protection of animal welfare as well as authorities of the Bundeswehr (Gz 42-34-30-20/G03-18) and were in accordance with the German Animal Welfare Act (BGBl. I S. 1206, 1313; May 18th, 2006) and the European Council Directive 2010/63/EU (September 22nd, 2010). Prior to experimentation, animals were allowed a 7-days acclimatization period to reduce stress from transport. On the day of the experiment, animals were anesthetized by intraperitoneal injection of a mixture containing ketamine (75 mg/kg body weight, b.w., Ketavet 100 mg/mL, zoetis Deutschland GmbH, Berlin, Germany) and xylazine (10 mg/kg b.w., Xylasel 20 mg/mL, Selectavet Dr. Otto Fischer GmbH, Weyarn-Holzolling, Germany). Sufficient depth of anesthesia during the experiment was determined by checking the hind-paw withdrawal reflex. If reflexes were evident, an additional injection with 2/3 of the initial anesthesia dose was administered intraperitoneally. For SM exposure, animals were positioned back facing upwards on a heated operating table and the fur in a 3 × 5 cm rectangle on the back was trimmed with a shearing machine, cutting the hair to about 2 mm length. Afterwards, the skin was cleaned with water, dried, and two adhesive strips both containing a pre-cut of 8 mm diameter each were attached at distant sites of the skin. Both areas were then exposed with 12.7, 31.8, 63.6, 95.4 or 127 mg of neat SM (8 M) for 1 h. Each concentration was applied to three animals (*n* = 3 per concentration). As a negative control, one animal was exposed the same way to 100 µL EtOH for 1 h. The exposed skin areas were covered with plastic caps after administration of SM or EtOH to prevent evaporation. After 1 h, the plastic caps were removed, and the skin was decontaminated using RSDL. While still under anesthesia, animals were then euthanized by cardiac puncture. EDTA blood was collected centrifuged (20 min, 4 °C, 500 RCF) and plasma was isolated for MS analysis of adducted rat serum albumin (RSA, see Plasma sample preparation and μLC-ESI MS/HRMS analysis of C(-HETE)PY). The SM-exposed skin areas were taken using 8 mm punches and stored at 4 °C until further processing for DNA- and CK-adduct analysis.

### µLC-ESI MS/HRMS system

For the investigation of alkylated proteins and DNA micro liquid chromatography-electrospray ionization high-resolution tandem-mass spectrometry (µLC-ESI MS/HRMS) measurements were performed using a hybrid quadrupole time-of-flight mass spectrometer (TT5600^+^, ABSciex, Darmstadt, Germany) online coupled to a microLC 200 pump (Eksigent Technologies LLC, Dublin, CA, USA) combined with an HTX-xt DLW autosampler (CTC Analytics, Zwingen, Switzerland) equipped with a 20 µL sample loop (Sunchrom, Friedrichsdorf, Germany). The sample tray was kept at 15 °C. The entire system was controlled by the Eksigent 4.2 (Eksigent Technologies LLC) and the Analyst TF 1.7.1 (ABSciex) software. All separations targeting either the alkylated CK-derived tetrapeptide TC(-HETE)PS or the albumin-derived alkylated tripeptide C(-HETE)PY or the DNA-derived alkylated bases HETE-Gua, HETE-Ade and Gua-ETE-Gua were carried out at 45 °C on an ACQUITY UPLC HSS T3 column (C18, 50 mm × 1.0 mm I.D., 1.8 μm, 100 Å, Waters, Eschborn, Germany) protected by a security guard ultra-cartridge (C18-peptide, Phenomenex, Aschaffenburg, Germany). Binary mobile phase gradients (see below) were used consisting of solvent A (0.05% v/v FA) and solvent B (acetonitrile/H_2_O 80:20 v/v, 0.05% v/v FA).

### Skin sample preparation and µLC-ESI MS/HRMS analysis of TC(-HETE)PS

Samples of human and rat skin ex vivo exposed to SM as well as rat skin obtained from one skin biopsy per animal from the in vivo experiments were homogenized (Sect. Homogenization of skin, lysis, and determination of protein concentration). CK was purified by IMS and digested by ProtK (Sect. Purification of CK from skin lysates by IMS and proteolysis) before MS/HRMS analysis. For µLC-ESI MS/HRMS analysis of the alkylated tetrapeptide TC(-HETE)PS the following gradient of solvent A and solvent B (Sect. μLC-ESI MS/HRMS system) was applied at a flow of 30 µL/min: *t* [min]/B [%]: 0/0; 11/35; 11.5/95; 13.5/95; 14/0; 15/0 at 45 °C including an initial equilibration time of 5 min. Productions of protonated TC(-HETE)PS ([M + H]^+^: *m/z* 511.2) and protonated d_3_-atropine ([M + H]^+^ *m/z* 293.1) were monitored using the following MS settings: curtain gas (CUR) 30 psi (2.1·10^5^ bar), heater gas (GS1) 40 psi (2.8·10^5^ bar), turbo ion spray gas (GS2) 50 psi (3.4·10^5^ bar), temperature (TEM) 200 °C, ion spray voltage floating (ISVF) 5.5 kV, ion release delay (IRD) 67 ms, accumulation time 300 ms, ion release width (IRW) 25 ms, collision energy (CE) 45 V (alkylated tetrapeptide) and 42 V (d_3_-atropine), collision energy spread (CES) 5 V, and declustering potential (DP) 60 V.

### Skin sample preparation and µLC-ESI MS/HRMS analysis of HETE-Gua, HETE-Ade and Gua-ETE-Gua

One skin biopsy per animal was used to extract DNA. The skin biopsy was trimmed to 25 mg by removing adherent fat tissue. DNA was extracted using the QIAamp DNA Mini Kit from Qiagen (Hilden, Germany) according to the manufacturer´s protocol. In brief, specimens were manually cut into small pieces, transferred into a 1.5 mL reaction vial, and immersed in 180 µL ATL buffer (Qiagen). Then, 20 µL ProtK solution (20 mg/mL in 50 mM NH_4_HCO_3_) was added and the samples were incubated at 56 °C for 4 h. After centrifugation, 4 µL RNase A (100 mg/mL, Qiagen) was added and samples were further incubated for 2 min at RT. AL buffer (200 µL, Qiagen) was added to the sample and incubated at 70 °C for 10 min. After adding 200 µL EtOH, the mixture was vortexed and applied to a QIAamp Mini spin column (Qiagen) and centrifuged for 1 min at 6,000 RCF. The eluate was discarded, and the column was washed twice with 500 µL washing buffer (Qiagen) each. The DNA was eluted by the addition of 200 µL buffer AE (Qiagen) to the column and subsequent centrifugation at 6,000 RCF for 1 min. The DNA eluate was digested by adding of 3.9 µL of 10 × digestion master mix consisting of 17 µL of 630 mM Tris buffer, pH 8, and 63 mM MgCl_2_, 2 µL benzonase (10 U/µL) and 20 µL DNAse (2 mg/mL). Samples were incubated at 37 °C for 2 h. Afterwards, 1.3 µL NaOAc (1 M, pH 7.8), 0.5 µL AP (10 U/µL) and 0.5 µL PDE (0.5 U/µL) were added and the mixture was incubated for 2 h at 37 °C. The incubated samples were transferred into an UF-device with a MWCO of 10 kDa and centrifuged for 10 min at 10,000 RCF and 15 °C. Then, 100 µL water was added followed by centrifugation (10 min, 10,000 RCF, 15 °C). The filtrate was heated to 90 °C for 20 min. For subsequent µLC-ESI MS/HRMS analysis, an aliquot was diluted 1:3 with d_3_-atropine solution (3 ng/mL, in 0.5% v/v FA). Alkylated nucleotides were separated at 50 µL/min applying a gradient of solvent A and B (Sect. μLC-ESI MS/HRMS system): *t* [min]/B [%]: 0/2; 3/60; 4.98/60; 5.0/2; 6.0/2 at 60 °C including an initial equilibration time of 3 min with 100 µL/min. Protonated HETE-Gua ([M + H]^+^ *m/z* 256.1), HETE-Ade ([M + H]^+^ *m/z* 240.1), Gua-ETE-Gua ([M + H]^+^ *m/z* 389.1) and d_3_-atropine ([M + H]^+^ *m/z* 293.1) were monitored in product ion scan mode applying the following MS settings: CUR 30 psi (2.1·10^5^ bar), GS1 50 psi (3.4·10^5^ bar), GS2 50 psi (3.4·10^5^ bar), TEM 300 °C, ISVF 5.0 kV, IRD 67 ms, accumulation time 50 ms, IRW 25 ms, CE 22 V (HETE-Gua), 20 V (HETE-Ade), 25 V (Gua-ETE-Gua), CES 5 V (HETE-Gua and HETE-Ade), 10 V (Gua-ETE-Gua) and DP 65 V.

### Plasma sample preparation and µLC-ESI MS/HRMS analysis of C(-HETE)PY

Rat plasma (100 µL) obtained from the in vivo experiments (Sect. In vivo animal study) was mixed with 50 mM NH_4_HCO_3_ (300 µL, pH 8) and ProtK solution (100 µL, 20 mg/mL in 50 mM NH_4_HCO_3_). After incubation at 50 °C for 4 h and UF (10 min, 15 °C, 10,000 RCF), samples were diluted 1:3 with d_3_-atropine solution (3 ng/mL in 0.5% v/v FA). For µLC-ESI MS/HRMS analysis the gradient of solvent A and solvent B (Sect. μLC-ESI MS/HRMS system) at 40 °C was as follows: *t* [min]/B [%]: 0/2; 11/30; 11.5/95; 13.5/95; 14/2; 15/2 with a flow of 30 µL/min including an initial equilibration time of 5 min with 30 µL/min and starting composition (2% v/v B). The mass spectrometer operated in the product ion scan mode monitoring product ions of the protonated, alkylated, RSA-derived tripeptide C(-HETE)PY ([M + H]^+^: *m/z* 486.2) and protonated d_3_-atropine using the following parameters: CUR 30 psi (2.1·10^5^ bar), GS1 40 psi (2.8·10^5^ bar), GS2 50 psi (3.4·10^5^ bar), TEM 200 °C, ISVF 5.5 kV, IRD 67 ms, accumulation time: 300 ms, IRW 25 ms, CE 35 V, CES 5 V, and DP 60 V.

## Results and discussion

### Detection of CK from rat and human skin by Western blot analysis

**Fig. 1:**
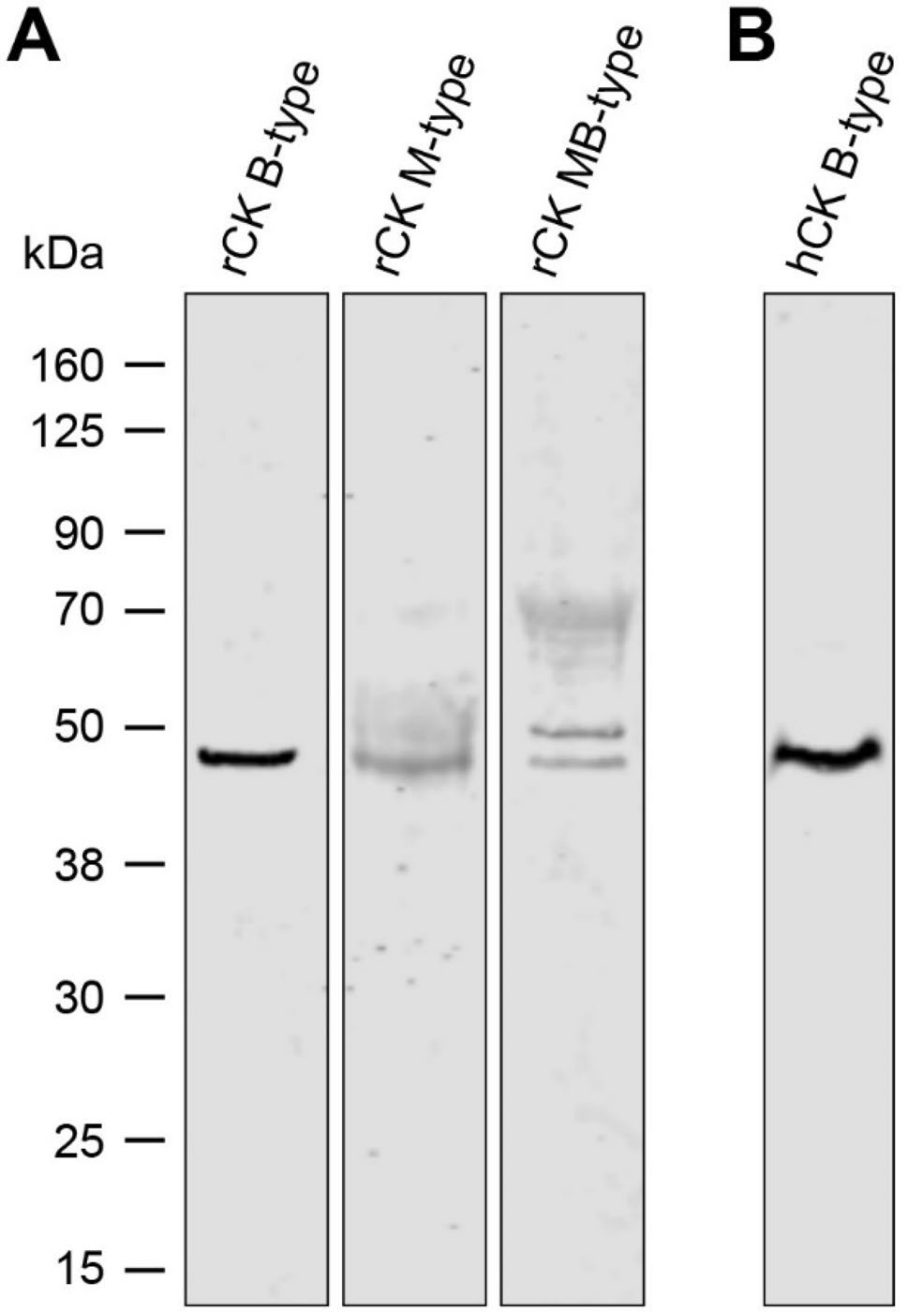
Western blot analyses of creatine kinase (CK) in ex vivo skin samples. **a** Screening for the three different isoenzymes of CK in rat skin using monoclonal anti-CK antibody targeting either the B-, M- or MB-type isoform. The CK B-type monomer with a molecular weight of approximately 43 kDa was found as the most abundant isoenzyme in rat skin. **b** Expression of the B-type isoform was also detected in human skin lysates

The molecular weight of the monomeric CK is approximately 43 kDa with slight variations between diverse species (Perryman et al. 1983). Western blot analysis under reducing conditions using antibodies directed against either the M-, B-, or MB-type of CK revealed that the B-type CK was predominately expressed in the rat (Fig. 1a) as well as in human skin (Fig. 1b). These findings are in accordance with the results presented by Schlattner et al. describing B-type CK as the most prominent isoform in murine skin (Schlattner et al. 2002) and those of Lenz et al. reporting on the expression of B-type CK in human skin (Lenz et al. 2005).

### Detection of CK alkylation in rat skin ex vivo

**Fig. 2:**
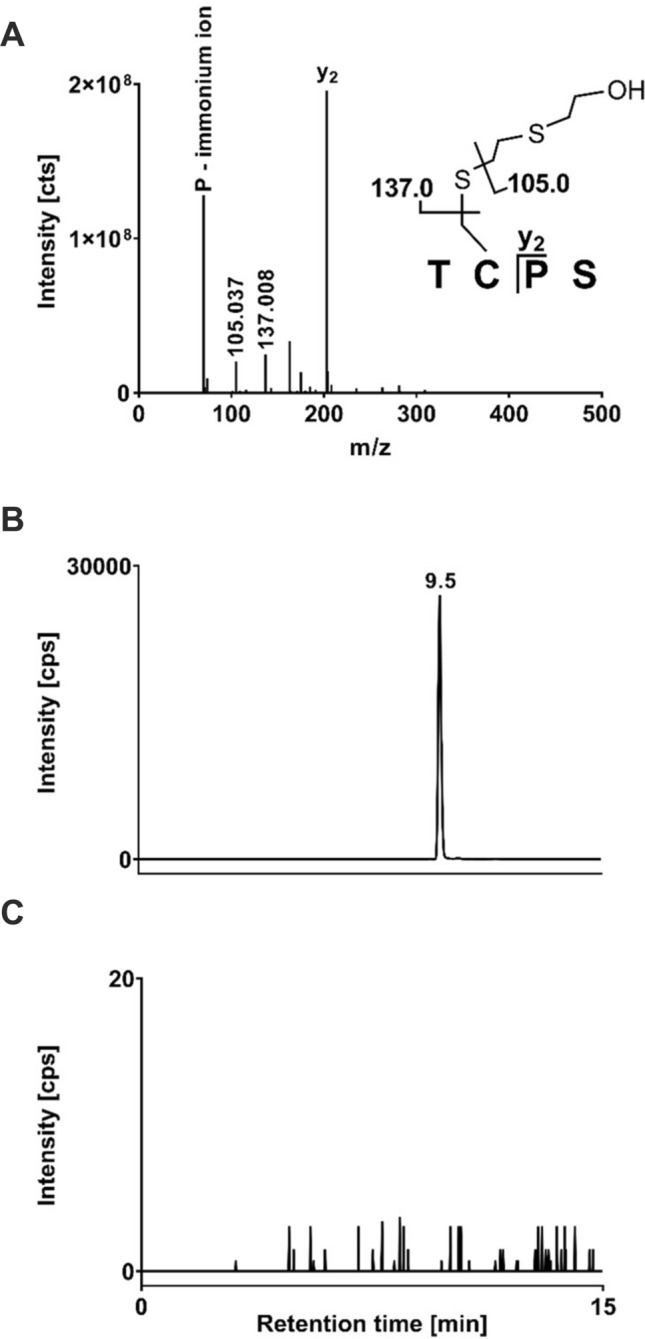
µLC-ESI MS/HRMS analyses of TC(-HETE)PS from rat skin exposed to SM. Ratskin was lysed, CK was extracted by IMS and subjected to proteolysis with ProtK. **a** MS/HRMS spectrum of the alkylated CK-derived tetrapeptide TC(-HETE)PS (*m/z* 510.896, single protonated) extracted from the µLC-ESI MS/HRMS run peak depicted in Figure part B. **b** Extracted ion chromatogram (XIC) of TC(-HETE)PS derived from rat skin exposed to SM. For reasons of clarity only the trace of the product ion at *m/z* 137.008 ± 0.005 is illustrated. **c** XIC of TC(-HETE)PS of a prepared rat skin blank sample not exposed to SM showing no interferences

Based on the positive Western blot results, rat skin was exposed to 12.7 mg SM (10 µL of neat SM applied on a surface of approximately 50 mm^2^) ex vivo and analyzed for HETE-CK adduct formation. As reported previously by our group, SM alkylates the reactive Cys^282^ residue in human CK (Lüling et al. 2020). However, after lysis of SM-exposed rat skin samples and direct subsequent proteolysis with ProtK and analysis by µLC-ESI MS/HRMS the expected alkylated CK-derived tetrapeptide TC(-HETE)PS was not detected. It was assumed that the failed detection was due to the low CK concentrations in the prepared samples, which were insufficient for MS-analysis without any concentration step. Therefore, we developed an IMS method well-suited for the extraction and enrichment of CK from skin lysates using the rabbit monoclonal anti-CK B-type antibody. After application of the IMS procedure, the expected alkylated CK-derived biomarker tetrapeptide was detected and identified by µLC-ESI MS/HRMS (Fig. 2a, b). Product ions detected were the same as already described and assigned recently (Lüling et al. 2020). Corresponding analysis of blank rat skin not exposed to SM did not show any interferences (Fig. 2c). The technical lower limit of detection (LOD), describing the lowest amount of SM used to expose rat skin biopsies (8 mm diameter corresponding to 50 mm^2^ skin area) ex vivo that still allowed the µLC-ESI MS/HRMS detection of the alkylated tetrapeptide biomarker, was estimated by dose–response experiments and found to correspond to 127 µg SM.

### Detection of CK alkylation in human skin ex vivo

The primary structure of rat (NCBI Reference Sequence NP_036661.3) and human CK B-type (NCBI Reference Sequence: NP_001814.2) exhibit a 100% identity as determined by the Constraint-based Multiple Alignment Tool (COBALT) (Papadopoulos and Agarwala 2007), Therefore, we also expected the formation of the CK-adduct in human skin. Accordingly, we succeeded in the detection of the biomarker TC(-HETE)PS in samples from human skin exposed to SM ex vivo. The corresponding MS/HRMS spectrum was identical to that obtained from rat CK (Fig. 2a) thus confirming the identity of the biomarker. Therefore, we concluded that CK, in general might represent a novel and valuable biomarker of SM exposure in humans and rat.

### In vivo animal study

Results discussed above suggest the HETE CK-adduct as a marker protein not only for ex vivo but also for i*n vivo* exposure scenarios. The adduct-derived tetrapeptide might represent a beneficial marker to document the exposure of a defined area on the skin (local biomarker) in contrast to marker proteins such as HETE-HSA (Noort et al. 1999; Gandor et al. 2015; John et al. 2016; Steinritz et al. 2016), HETE-hemoglobin (Noort et al. 1996; Black et al. 1997) or HETE-DNA from blood cells (Dębiak et al. 2011; Zubel et al. 2018, 2019) that provide evidence for systemic exposure. Therefore, an animal study investigating rats exposed to different concentrations of SM was carried out monitoring the different local (HETE-CK and HETE-DNA from skin cells) and the systemic adducts (HETE-RSA). Animals were exposed at two distant sites with identical doses of SM allowing analysis of HETE-DNA-adducts from one site and of HETE-CK at the other site.

#### Detection of HETE-CK adducts in rat skin in vivo

**Fig. 3:**
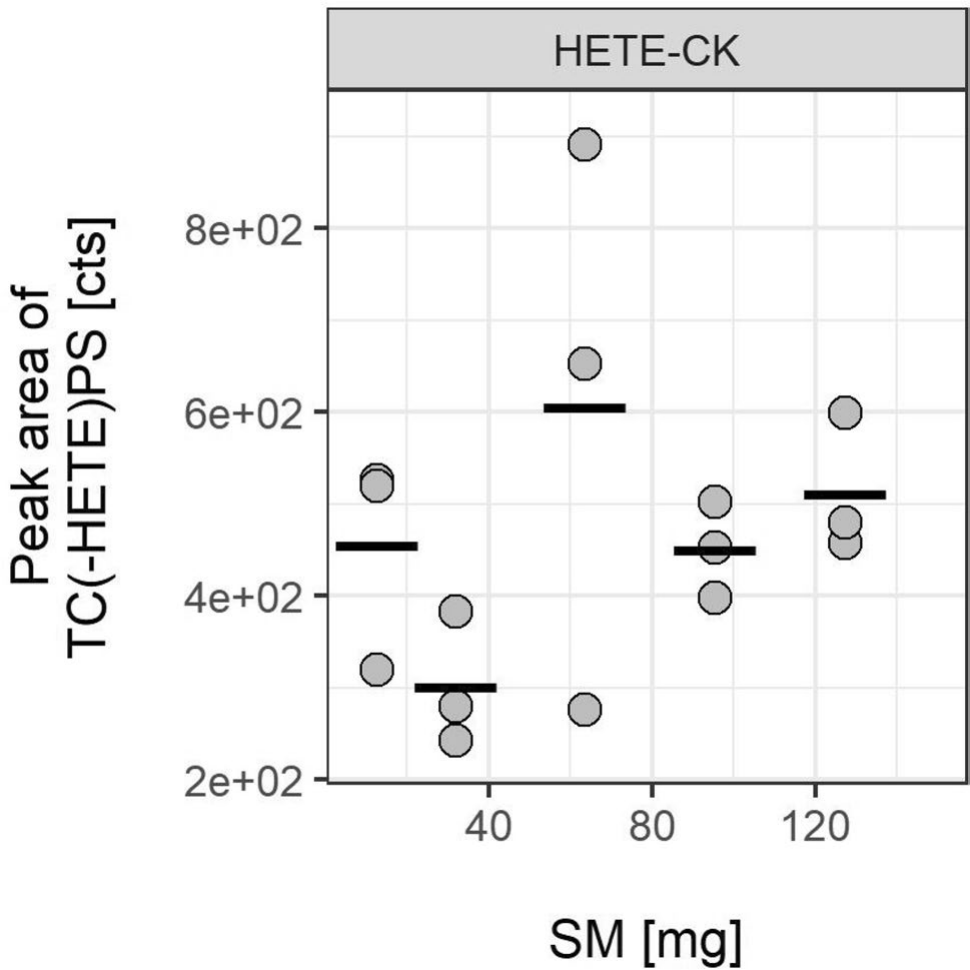
Peak areas of the alkylated CK-derived tetrapeptide TC(-HETE)PS obtained from µLC-ESI MS/HRMS analyses of rat skin exposed to SM in vivo. Skin samples were prepared postmortem by lysis, IMS to extract CK B-type, proteolysis of the protein with ProtK and final UF. An adduct formation was observed in each group, qualitatively proving the exposure. In vivo exposure experiments were carried out in triplicates (*n* = 3). Data points represent the individual values related to each animal and the bars provide the means of triplicate measurements

All SM doses applied resulted in the local formation of the HETE-CK adduct (Fig. 3). However, the correlation between the SM amount applied and the peak area of the TC(-HETE)PS biomarker extracted from a defined area of the skin (50 mm^2^) was rather less obvious. Most presumably this effect might be due to rapid and extensive diffusion processes of SM in the perfused skin of the living rat after challenging a prefixed skin area. Moreover, an unknown amount of the applied SM may have been absorbed in subdermal fat tissue thereby bypassing a reaction with epidermal CK. However, the CK-derived tetrapeptide served as a reliable qualitative local biomarker of the skin in all animals independent of the SM dose applied thus underlining the high chemical reactivity with CK and its suitability as a marker protein.

#### Detection of HETE-DNA adducts in rat skin in vivo

**Fig. 4:**
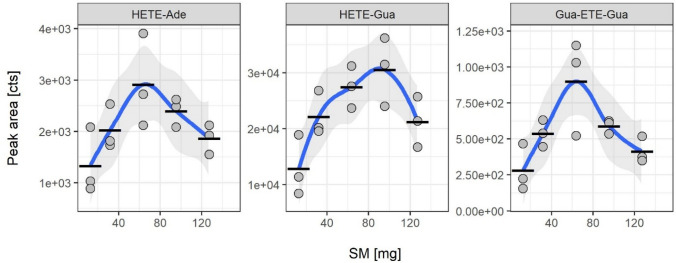
Peak areas of the alkylated DNA-derived nucleotides HETE-Ade, HETE-Gua and Gua-ETE-Gua obtained from µLC-ESI MS/HRMS analyses of rat skin exposed to SM in vivo. Ratskin specimens were trimmed to 25 mg. Samples were subsequently prepared by ProtK, DNAse, heat treatment, and UF prior to µLC-ESI MS/HRMS analysis. In vivo exposure experiments were carried out in triplicate (*n* = 3). Data points represent the individual values related to each animal and the bars provides the means of triplicate measurements. The ribbon indicates the 95% confidence interval of the linear curve fit

The digested DNA was analyzed to monitor HETE-Gua, HETE-Ade and the crosslinked Gua-ETE-Gua biomarkers (Zubel et al. 2019). MS/HRMS spectra (data not shown) confirmed the identity of the adducts corresponding to data published recently (Zubel et al. 2019). The most prominent product ion produced by the three biomarkers was the HETE-moiety (*m/z* 105.037). Accordingly, this ion was used to follow the relative concentration profiles of the markers depending on the dose of SM administered. The dose–response relationship of SM-DNA adduct formation in rat skin followed a reverse parabola shape (Fig. 4).

The decrease of the peak areas of nucleic acid-adducts after reaching a maximum at a certain SM concentration might be indicative for an increasing extent of cross-links between the respective nucleobase and either other DNA-bases or proteins, or for the prolongation of the attached modification by SM-induced alkylation of an already attached HETE-moiety. However, more plausible it might also be assumed that the larger extent of DNA crosslinks caused by higher SM doses may impact the sample preparation procedure by potentially deteriorating extraction and digest, thus reducing the concentration of the analyte. This has also been observed by Moser et al*.* who reported difficulties arising from the presence of 30 µM SM and above in the detection of DNA strand breaks in peripheral blood leukocytes (Moser et al. 2004). Kehe et al. reported a similar effect when using a standard Comet assay to detect SM-induced DNA damages in a keratinocyte cell line and assumed increased DNA crosslinks that prevented migration in the electrical field (Kehe et al. 2009a). In an in vivo study, conducted by Zhang et al*.*, rabbits were dermally exposed to SM doses of 2–15 mg/kg b.w. and the formation of SM-DNA adducts in urine was investigated (Zhang et al. 2014). The authors successfully detected alkylation of the respective nucleotides at all SM doses, but the percentage of SM-DNA adducts after exposure to 15 mg/kg b.w. SM was significantly lower compared to the 5 mg/kg b.w. SM dose. Thus, although SM-DNA adducts are a valuable and frequently used tissue biomarker, its quantitative interpretation is limited when used in high dose exposure scenarios.

#### Detection of HETE-albumin adducts from rat plasma

**Fig. 5:**
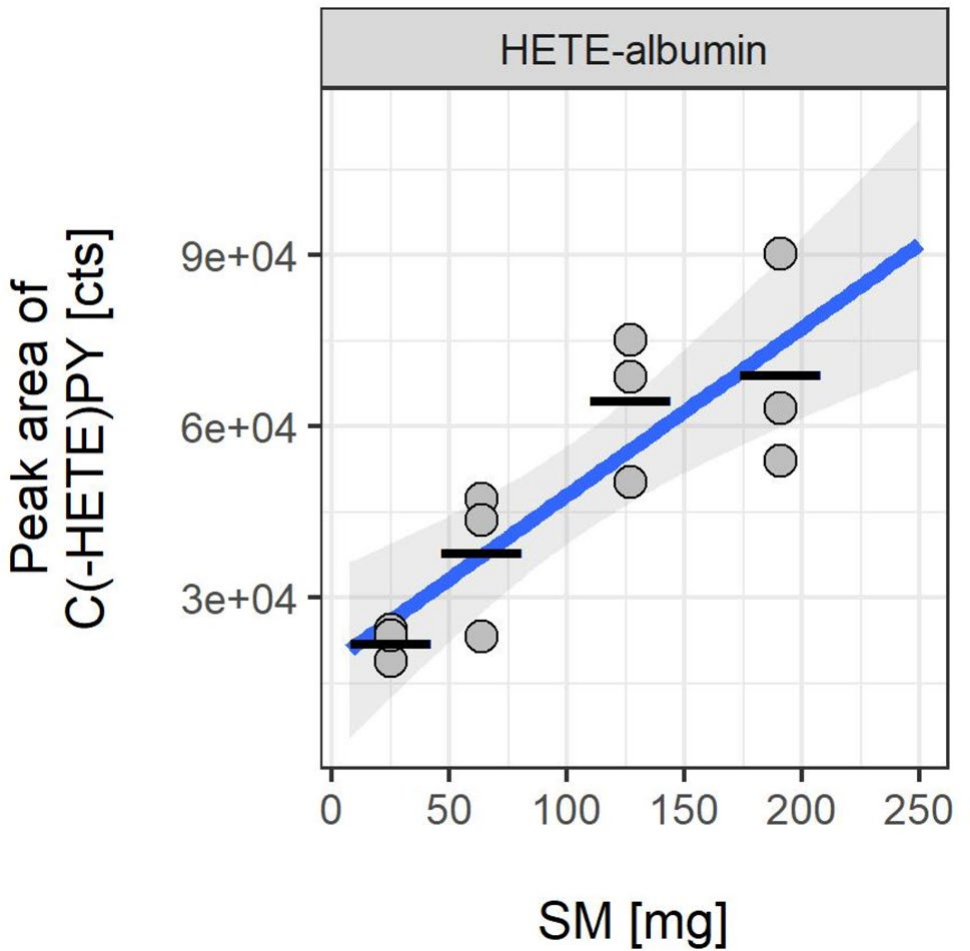
Peak areas of the alkylated RSA-derived tripeptide C(-HETE)PY obtained from µLC-ESI MS/HRMS analyses of rat plasma exposed to SM in vivo. Plasma samples were prepared by proteolysis with ProtK and UF. In vivo exposure experiments were carried out in triplicates (*n* = 3). Data points represent the individual values related to each animal and the bars provide the means of triplicate measurements. The ribbon indicates the 95% confidence interval of the linear curve

The formation of HETE-RSA adducts after the challenge of rats with SM has been described by Noort et al. (2008). They introduced the alkylated tripeptide C(-HETE)PY as a reliable in vivo biomarker produced by proteolysis of the RSA-adduct (Noort et al. 2008). Therefore, this analyte was also monitored in the present study to document a potential systemic uptake of SM. The peak areas of the biomarker in µLC-ESI MS/HRMS analysis correlated to the concentration of HETE-RSA present in vivo. In contrast to the findings for the local biomarkers derived from skin CK (Fig. 3) and skin DNA (Fig. 4) a concentration-dependent linear increase of the biomarker concentration was observed (Fig. 5). Blank plasma obtained from a rat not exposed to SM did not show any adduct and interferences in µLC-ESI MS/HRMS analysis. The dose–response effect was similar to the experiments performed by our group in vitro (Gandor et al. 2015; John et al. 2016). The evidence of the albumin-adducts in plasma proved that SM, even when applied at low doses and for only 1 h, has penetrated the skin and entered the circulation allowing its systemic distribution and adduct formation in the blood.

## Conclusion and outlook

Due to this experimental study, CK or rather Cys^282^ in CK B-type was identified as a new dermal target of low and local SM exposures. To our knowledge, it is also the first time that HETE-albumin adducts, and HETE-DNA adducts were monitored simultaneously in an in vivo animal study.

The verification of exposure was possible using the HETE-albumin-, -DNA-, and -CK-derived biomarker. Alkylation of albumin, representing a systemic biomarker, was observed already after exposure to 25 mg SM applied on rat skin, underlining the value and significance of HETE-albumin adducts for verification purposes. Alkylation of DNA nucleotides in skin tissue, representing a local biomarker, was also detected at all SM-doses. However, at SM doses > 63 mg (applied on an 8 mm^2^ skin area) the concentration of these biomarkers decreased. The correlation of the biomarker concentration to an estimated exposure dose may thus be hampered. The formation of HETE-CK adducts was proven already after 12.7 mg SM applied on an 8 mm^2^ skin area. Compared to HETE-DNA adducts, a loss of sensitivity at increasing SM doses was not evident. Hence, HETE-CK adducts may be well suited when used as local biomarker in high-dose exposures. Future studies are planned to investigate dose–response relationships in more detail.

B-type CK, derived from epidermal cells, was detected in blister fluid that were provoked by shear stress (Paavonen et al. 1988; Kiistala et al. 1989). Since blister formation occurs in SM-induced skin injury (Kehe et al. 2009b) with accompanying cell death of affected keratinocytes (Rosenthal et al. 1998), these cells may release alkylated CK into the blister fluid. Hence, CK might also be considered as an additional biomarker in such biological specimens.

## Abbreviations

Ade: Adenine
b.w.: Body weight
CE: Collision energy
CES: Collision energy spread
CK: Creatine kinase
CUR: Curtain gas
DNA: Deoxyribonucleic acid
DP: Declustering potential
DTT: Dithiothreitol
ETE: Ethylthioethyl-moiety
EtOH: Ethanol
ESI: Electrospray ionization
FA: Formic acid
GS1: Heater gas
GS2: Turbo ion spray gas
Gua: Guanine
HETE: Hydroxyethylthioethyl-moiety
HSA: Human serum albumin
IAA: Iodoacetamide
IMS: Immunomagnetic separation
IRD: Ion release delay
IRW: Ion release width
ISVF: Ion spray voltage floating
LOD: Lower limit of detection
MWCO: Molecular weight cut-off
µLC: Micro liquid chromatography
MS/HRMS: High-resolution tandem-mass spectrometry
MS/MS: Tandem-mass spectrometry
OPCW: Organisation for the Prohibition of Chemical Weapons
PAGE: Polyacrylamide gel electrophoresis
PBS: Phosphate buffered saline
RCF: Relative centrifugal field
RSA: Rat serum albumin
RSDL: Reactive skin decontamination lotion
RT: Room temperature
SM: Sulfur mustard, *bis*-(2-chloroethyl) sulfide
TEM: Temperature (as ion source setting)
UF: Ultrafiltration
